# A Novel Method for Carbendazim High-Sensitivity Detection Based on the Combination of Metamaterial Sensor and Machine Learning

**DOI:** 10.3390/ma15176093

**Published:** 2022-09-02

**Authors:** Ruizhao Yang, Yun Li, Jincun Zheng, Jie Qiu, Jinwen Song, Fengxia Xu, Binyi Qin

**Affiliations:** 1 Key Laboratory of Complex System Optimization and Big Data Processing, Guangxi Colleges and Universities, Yulin Normal University, Yulin 537000, China; 2 Optoelectronic Information Research Center, School of Physics and Telecommunication Engineering, Yulin Normal University, Yulin 537000, China; 3 School of Chemistry and Food Science, Yulin Normal University, Yulin 537000, China; 4 Research Center of Intelligent Information and Communication Technology, School of Physics and Telecommunication Engineering, Yulin Normal University, Yulin 537000, China; 5 School of Computer Science and Engineering, Yulin Normal University, Yulin 537000, China

**Keywords:** metamaterial sensor, mean shift, terahertz spectroscopy

## Abstract

Benzimidazole fungicide residue in food products poses a risk to consumer health. Due to its localized electric-field enhancement and high-quality factor value, the metamaterial sensor is appropriate for applications regarding food safety detection. However, the previous detection method based on the metamaterial sensor only considered the resonance dip shift. It neglected other information contained in the spectrum. In this study, we proposed a method for highly sensitive detection of benzimidazole fungicide using a combination of a metamaterial sensor and mean shift machine learning method. The unit cell of the metamaterial sensor contained a cut wire and two split-ring resonances. Mean shift, an unsupervised machine learning method, was employed to analyze the THz spectrum. The experiment results show that our proposed method could detect carbendazim concentrations as low as 0.5 mg/L. The detection sensitivity was enhanced 200 times compared to that achieved using the metamaterial sensor only. Our present work demonstrates a potential application of combining a metamaterial sensor and mean shift in benzimidazole fungicide residue detection.

## 1. Introduction

Due to its efficacy and affordability, benzimidazole fungicide has been used extensively to control crop pests. However, excessive usage of benzimidazole fungicide has resulted in its residue being found in foods including rice, wheat, corn, soybeans, etc. It directly threatens the health and safety of consumers [1,2]. Food products should be checked for benzimidazole fungicide before consumption to ensure food safety. Carbendazim is a benzimidazole fungicide widely used to prevent crop disease caused by fungi. The maximum residue limit of carbendazim in cereals is 0.5 mg/kg according to Chinese Standard GB2763-2021. Currently, chromatographic, biological, and spectroscopic techniques are the main methods for detecting pesticide residues. Chromatography is sensitive but requires tedious sample pretreatment [3,4,5,6,7]. The biological method can detect pesticide residues rapidly, but it is not universal [8,9,10,11]. The spectroscopic method, such as Raman spectroscopy and infrared spectroscopy, is straightforward, quick, safe, and nondestructive [12,13,14,15,16,17]. Gong et al. [18] reported a surface-enhanced Raman scattering (SERS) method for detecting pesticide residues on fruit peels. SERS was achieved by applying silver nanoparticles (Ag NPs) to the surface of the tape after analyte collection. The limit of detection was 0.0225 mg/kg. Madianos et al. [19] developed a pesticide gas sensor based on self-assembled nanoparticle networks and four different polymer coatings. The sensors could detect chlorpyrifos concentrations as low as 0.7 ppb. Chen et al. [20] developed an Ag–nanocellulose fiber SERS substrate (Ag@NCF substrate) to detect hazardous residues. The sensitive Ag@NCF substrates achieved ppb-level detection of pesticides. The spectroscopic method has garnered more and more interest in detecting pesticide residues.

The terahertz (THz) spectrum, lying between 0.1 THz and 10 THz, is a new spectroscopic method. In molecular and biomolecular systems, the absorption of the THz spectrum is dominated by the excitation of intramolecular and intermolecular vibrations. It is significant for molecule and biomolecule detections. Thus, THz–TDS is regarded as a promising chemical and biological substance detection method [21,22,23,24]. In the field of pesticide detection, the pesticides imidacloprid, acetamiprid, triadimefon, carbendazim, thiamethoxam, and thiabendazole have been inspected by THz–TDS [25,26,27,28,29]. Nevertheless, the limited sensitivity prohibits THz–TDS from being widely used in pesticide detection.

Metamaterials are periodic artificial electromagnetic media structured on a subwavelength scale and exhibit characteristics not found in the natural world [30]. Because of its localized electric-field enhancement and high-quality factor value, the metamaterial sensor is appropriate for applications concerning food safety detection. Xie et al. [31] used a metamaterial sensor to detect traces of kanamycin sulfate. Compared to the lowest detectable concentration on bare silicon, the results reveal a $10^{10}$ improvement using metamaterial sensors. Qin et al. [32] used a circle-slit-shaped metasurface for tetracycline hydrochloride detection. The sensitivity increased $10^{5}$ times compared to detecting on a silicon substrate alone. Xu et al. [29] reported the chlorpyrifos-methyl-sensing performances of the THz metamaterial sensor. An Ohm ring metamaterial sensor for carbendazim detection was experimentally demonstrated by Qin [33]. However, the abovementioned detection method only considered the resonance dip shift. Much information contained in the spectrum was neglected. Machine learning methods have been employed to analyze the absorption of the THz spectrum in recent years. Yang et al. [34] employed popular machine learning methods, including the convolutional neural network, linear discriminant analysis (LDA), and the support vector machine, to classify the geographic origin of coffee beans. Silva et al. [35] presented an analytical method based on THz–TDS and partial least-squares regression to quantify mebendazole polymorphs (forms A, B, and C) in pharmaceutical raw material. The detection limits for polymorphs A, B, and C were 2.7–4.3% *w*/*w*, 2.9–4.0% *w*/*w*, and 2.4–3.1% *w*/*w*, respectively. To classify 20 amino acids, Wang et al. [36] designed a convolutional neural network calibrated by efficient channel attention. On two test datasets, the designed network achieved an accuracy of 99.9% and 99.2%. In our recent work, we proposed a WGAN-ResNet method, which combined two deep learning networks, the Wasserstein generative adversarial network (WGAN) and the residual neural network (ResNet), to detect carbendazim based on terahertz spectroscopy [37]. The outcomes demonstrated that the machine learning method could extract spectral features successfully. Thus, combining the metamaterial sensor and machine learning is a potential method to detect benzimidazole fungicide residue with high sensitivity.

In this paper, we present a highly sensitive method for detecting carbendazim using a metamaterial sensor and machine learning. We designed a metamaterial sensor, in which the unit cell consisted of a cut wire (CW) and two split-ring resonators (SRRs). The mechanism for resonance and the detection sensitivity were analyzed by simulation. Then, the amplitude transmission spectrum was measured without/with the carbendazim–ethyl alcohol solution mixture on the metamaterial sensor. We observed that two resonance dips shifted to a lower frequency with increased carbendazim concentrations. To improve the detection sensitivity, we employed an unsupervised machine learning method, mean shift, to analyze the amplitude transmission spectrum. The results demonstrate that the metamaterial sensor combined with the mean shift method could highly improve the device sensitivity.

## 2. Materials and Methods

### 2.1. Samples Preparation

The purity of the carbendazim powder was 98%. The purity of the anhydrous alcohol was 99%. They were bought from Adamas Co., Ltd. These chemicals were used without purification. We employ a magnetic stirrer to ensure that the carbendazim and anhydrous alcohol mixture was stirred consistently. Two duplicates were made for each concentration of the carbendazim–ethyl alcohol mixture solutions, which ranged in concentration from 0.5 mg/L to 100 mg/L (0.5 mg/L, 2 mg/L, and 100 mg/L).

### 2.2. Metamaterial sensor Structure and Simulation

**Figure 1:**
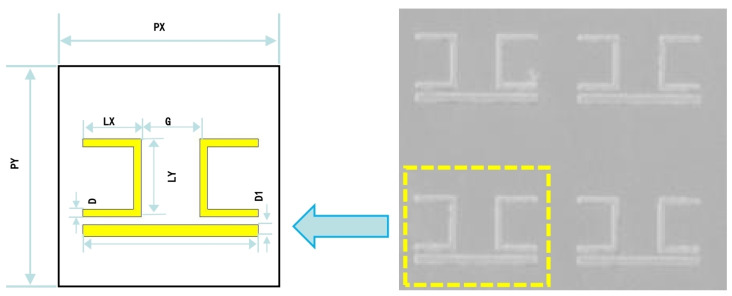
SEM image of the fabricated metamaterial sensor.

The metamaterial sensor was fabricated using a surface micromachining process. First, a layer of photoresist was used to form the CW and SRR structure. After the ultraviolet exposure and development process, the CW and SRR pattern was formed on the high-resistance silicon substrate. Then, a layer of the aluminum film was deposited on the surface. Finally, a lift-off process formed the metal CW and SRR structure. A scanning electron microscope (SEM) image of the fabricated metamaterial sensor is shown in Figure 1.

A CW and two SRRs were the components of the unit structure, which was deposited on a silicon substrate with high resistance. We call the fabricated metamaterial sensor CWSRRs. Both the CW and SRRs were aluminum. The following are the sensor’s structural parameters: PX= 120 μm, PY= 120 μm, LX = 40 μm, LY= 30 μm, D= 4 μm, D1= 6 μm, G= 30 μm. The aluminum and the high-resistance silicon substrate had thicknesses of 200 nm and 30 μm, respectively. We used the FITD method to simulate the metamaterial sensor CWSRRs. The simulation was implemented using the commercial full-wave simulation software CST2015 microwave studio. In the CWSRR simulation, we treated the high-resistance silicon substrate as a lossless dielectric with a dielectric permittivity of ε=11.9, and the DC conductivity of aluminum with $3.56\times 10^{7}$ S/m. The x and y directions were set as the unit cell boundary conditions, while the z direction was set as the open boundary condition.

### 2.3. Mean Shift

Mean shift is an unsupervised machine learning method that Fukunaga [38] proposed. The fundamental mean shift algorithm was generalized in the following two ways by Cheng [39]. One was to define the kernel function family. The other was to assign different weight coefficients to different sample points.

Given *n* data points $x_{i}$, i=1,...,n in the d-dimensional space $R^{d}$, the multivariate kernel density estimator with kernel K(x) and a symmetric positive definite d×d bandwidth matrix **H**, computed in the point x, was given by

(1) $$\hat{f}\left(x\right)=\frac{1}{n}\sum_{i=1}^{n}K_{H}\left(x-x_{i}\right)$$

where

(2) $$K_{H}\left(x\right)={\left|H\right|}^{-1/2}K\left(H^{-1/2}x\right)$$

The bandwidth matrix **H** was usually chosen in proportion to the identity matrix $H=h^{2}I$. The kernel density estimator was computed in the following manner:

(3) $$\hat{f}\left(x\right)=\frac{1}{nh^{d}}\sum_{i=1}^{n}K\left(\frac{x-x_{i}}{h}\right)$$

The kernel was satisfying

(4) $$K\left(x\right)=c_{k,d}k\left({∥x∥}^{2}\right)$$

where the k(x) was called as the profile of kernel, only for x≥0. The normalization constant $c_{k,d}$, which makes K(x) integrate to one, was assumed to be strictly positive.

Employing the profile notation, the density estimator can be rewritten as

(5) $${\hat{f}}_{h,K}\left(x\right)=\frac{c_{k,d}}{nh^{d}}\sum_{i=1}^{n}k\left({∥\frac{x-x_{i}}{h}∥}^{2}\right)$$

The density gradient estimator was obtained as the gradient of the density estimator by exploiting the linearity of Equation (5)

(6) $$\hat{\nabla }f_{h,K}\left(x\right)\equiv \nabla {\hat{f}}_{h,K}\left(x\right)=\frac{2c_{k,d}}{nh^{d+2}}\sum_{i=1}^{n}\left(x-x_{i}\right)k^{\prime }\left({∥\frac{x-x_{i}}{h}∥}^{2}\right)$$

Moreover, a function g(x) was defined

(7) $$g\left(x\right)=-k^{\prime }\left(x\right)$$

The derivative of the kernel profile *k* exists for all x∈[0,∞), except for a finite set of points. Using g(x) for profile, the kernel G(x) was defined as

(8) $$G\left(x\right)=c_{g,d}g\left({∥x∥}^{2}\right)$$

where $c_{g,d}$ was the corresponding normalization constant. Introducing g(x) into Equation (6) yields

(9) $$\begin{array}{cc} \; & \hat{\nabla }f_{h,K}\left(x\right)\\ \; & =\frac{2c_{k,d}}{nh^{d+2}}\sum_{i=1}^{n}\left(x_{i}-x\right)g\left({∥\frac{x-x_{i}}{h}∥}^{2}\right)\\ \; & =\frac{2c_{k,d}}{nh^{d+2}}\left[\sum_{i=1}^{n}g\left({∥\frac{x-x_{i}}{h}∥}^{2}\right)\right]\left[\frac{\sum_{i=1}^{n}x_{i}g\left({∥\frac{x-x_{i}}{h}∥}^{2}\right)}{\sum_{i=1}^{n}g\left({∥\frac{x-x_{i}}{h}∥}^{2}\right)}-x\right] \end{array}$$

From the Equation (5), the first term was proportional to the density estimate at x computed with the kernel *G*

(10) $${\hat{f}}_{h,G}\left(x\right)=\frac{c_{g,d}}{nh^{d}}\sum_{i=1}^{n}g\left({∥\frac{x-x_{i}}{h}∥}^{2}\right)$$

The second term was the mean shift vector

(11) $$M_{h,G}\left(x\right)=\frac{\sum_{i=1}^{n}x_{i}g\left({∥\frac{x-x_{i}}{h}∥}^{2}\right)}{\sum_{i=1}^{n}g\left({∥\frac{x-x_{i}}{h}∥}^{2}\right)}-x$$

The mean shift pseudo code is given in Algorithm 1.
**Algorithm 1** Mean shift**Input:** data, bandwidth**Output:** labels 1: Choose a point at random as the initial center point.  2: Find all points whose distance from the center point is less than the bandwidth, denoted as set S, and consider these points as belonging to cluster C.  3: Calculate the vectors starting from the center point to each element in the set S. Add these vectors to get the mean shift $M_{h,G}\left(x\right)$. 4: Calculate the center point:Centerpoint=Centerpoint+$M_{h,G}\left(x\right)$. 5: Repeat steps 2–4 until $∥M_{h,G}\left(x\right)∥\leq \varepsilon$ (ε is a threshold). 6: If the distance between the center point of cluster C converges and the center point of other clusters $C_{i}$ is less than the threshold, the two clusters are merged. Otherwise, list C as a separate new cluster. 7: Repeat steps 1–6 until all points have been marked. 8: **return** labels


### 2.4. Spectral Measurements

**Figure 2:**
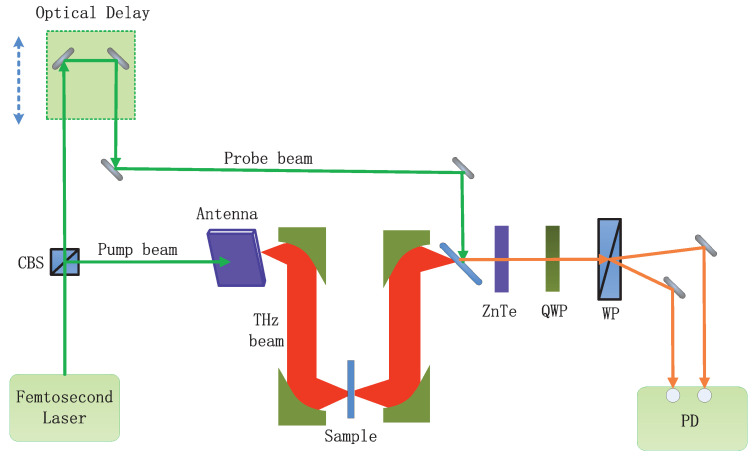
The schematic diagram of THz–TDS system.

All measurements were taken with a time-domain spectroscopy (THz–TDS) system operating in transmission mode at normal incidence. A schematic diagram of the THz–TDS system is shown in Figure 2. A cubic beam splitter (CBS) separated a femtosecond laser beam into a pump beam and a probe beam. The pump beam was illuminated on a photoconductive antenna to elicit the THz beam. THz detection was accomplished using an electro-optic ZnTe crystal, a quarter-wave plate (QWP), a Wollaston prism (WP), and a set of balanced photodiodes (PD). The spot diameter of the THz beam focused on the sample was approximately 4 mm.

A carbendazim–ethyl alcohol solution sample was doped on the metamaterial sensor for sample measurement. The metamaterial sensor was thoroughly cleaned with deionized water following the test, and it was then dried in a dry airflow. Each measurement was averaged from ten scans to reduce the random error. All tests were conducted in a dry container with a relative humidity of less than 1% (±0.1%), and at 25 °C (±0.1 °C).

## 3. Results and Discussion

### 3.1. Analysis of Simulation Results

**Figure 3:**
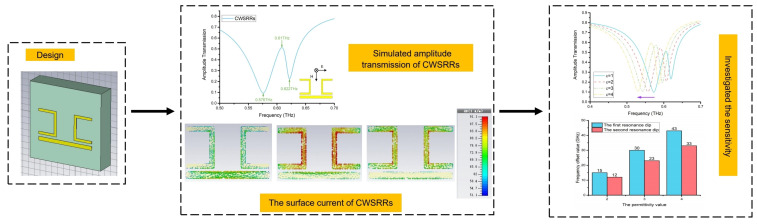
The flow chart for the simulation process.

Figure 3 depicts the simulation process flow chart. Using CST2015, we designed the metamaterial sensor CWSRRs. The amplitude transmission of CWSRRs was then obtained. Simultaneously, the mechanism of resonance was investigated using the surface current. Finally, the sensitivity of the resonance dips was investigated.

**Figure 4:**
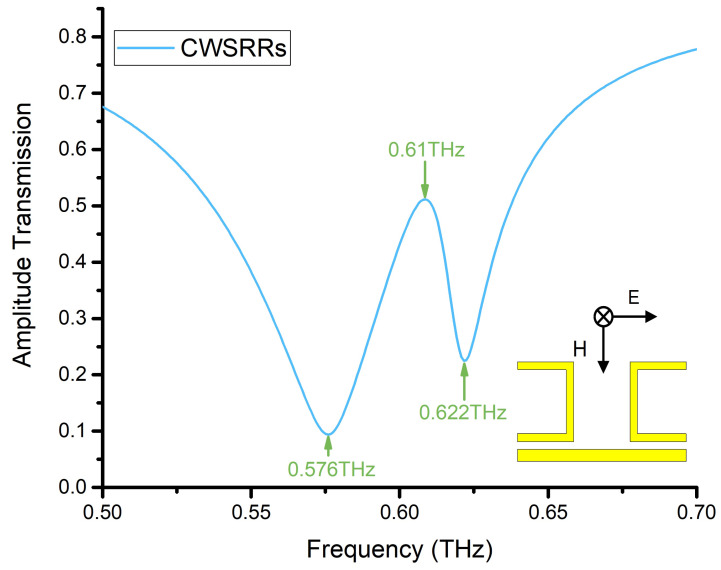
Simulated amplitude transmission of CWSRRs.

The simulated amplitude transmission of CWSRRs is shown in Figure 4. The resonance in CWSRRs displayed multimodal resonances, including two resonance dips at 0.576 THz and 0.622 THz, as well as a transmittance peak at 0.61 THz.

**Figure 5:**
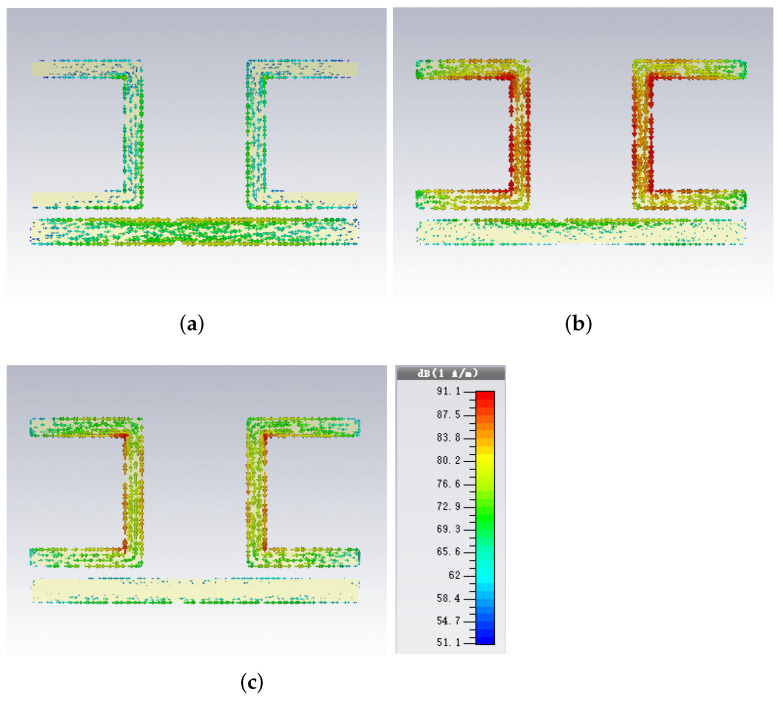
The surface current of CWSRRs at (**a**) 0.576 THz, (**b**) 0.61 THz, and (**c**) 0.622 THz.

For the first frequency resonance dip f= 0.576 THz, the surface current of CWSRRs is shown in Figure 5a. The surface current flows in the direction of the arrow. The various colors represent the magnitude of the surface current. Blue represents the smallest surface current, whereas red represents the largest surface current. We observed that the surface current was distributed on the CW and SRRs. The surface current on the CW was directly excited by the external field. Then, the field generated by the CW excited the surface current of SRRs through the near field. The color of the surface current on the CW was closer to red. This means that the surface current on the CW was larger than that on SRRs. Thus, the first resonance dip originated from electric dipole oscillations. For the second resonance dip f= 0.622 THz, the surface current of CWSRRs is shown in Figure 5c. The surface current was mainly distributed on SRRs. Compared with the first resonance dip, the surface current on SRRs was enhanced because the energy transferred from the CW to SRRs increased. Therefore, the second resonance dip was attributed to inductor–capacitor oscillations. For the transmittance peak at f= 0.61 THz, the surface current was distributed on the CW and SRRs, as shown in Figure 5b. The CW was directly excited by the external field as the bright mode. Additionally, the SRRs were in the dark mode, which cannot be directly excited by the external field. The dark mode was excited by the bright mode through the near-field coupling. The resonance peak occurred because of the absorption cancellation by the bright mode and the dark mode coherence and interference.

**Figure 6:**
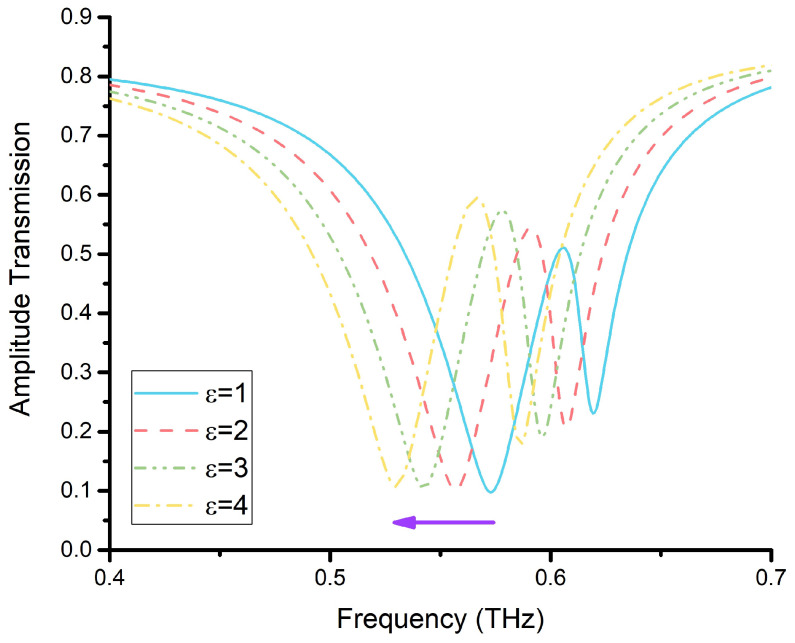
The transmission of CWSRRs with different permittivity values.

Furthermore, we investigated the sensitivity of the two resonance dips. The analyte’s thickness was set at 4 μm. We varied the permittivity from ε=1 to ε=4 with an incremental step of 1. The total electric capacity of the sensor was $C=C_{1}+C_{2}+C_{3}+C_{4}$ [40], $C_{1}$ was the electric capacity of the substrate, $C_{2}$ was the electric capacity between the substrate and the sensor structure, $C_{3}$ was the electric capacity of the sensor structure, and $C_{4}$ was the electric capacity between sensor structure and the analyte. As we know, the electric capacity C=εS/d. Thus, $C_{4}$ increased with the permittivity of the analyte that covered the sensor. The two resonance dips shifted to a low frequency as the permittivity of the analyte increased, as shown in Figure 6.

**Figure 7:**
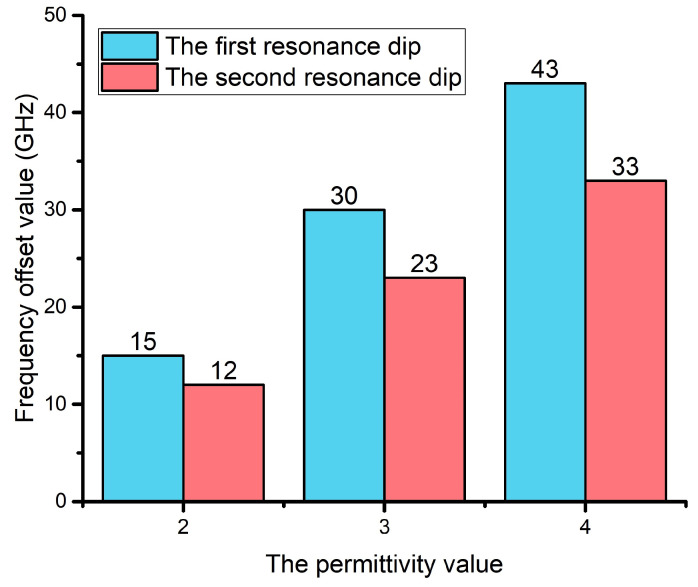
The frequency offset value of the two resonance dips.

Taking the permittivity ε=1 as a reference, the frequency offset value of the two resonance dips with different permittivities is shown in Figure 7. The frequency offset value was defined as the difference between the resonance frequency of the reference and the analyte. The X axis was the permittivity, and the Y axis was the frequency offset value. At the same permittivity value, the frequency offset value of the first resonance dip was more significant than the second resonance dip. With the permittivity increasing, the frequency offset value of the first resonance dip varied from 15 GHz to 43 GHz, and the second resonance dip changed from 12 GHz to 33 GHz. The sensitivity of the first and second resonance dip was 43 GHz/RIU and 33 GHz/RIU.

### 3.2. Analysis of Experiment Results

**Figure 8:**
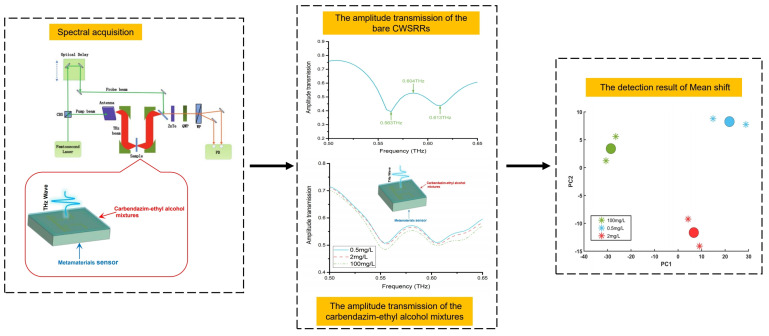
The flow chart for the experiment process.

Figure 8 shows the flow chart for the experiment process. The THz–TDS system obtained the amplitude transmission without/with the carbendazim–ethyl alcohol mixtures on CWSRRs. Then, the mean shift was employed to analyze the amplitude transmission spectra.

**Figure 9:**
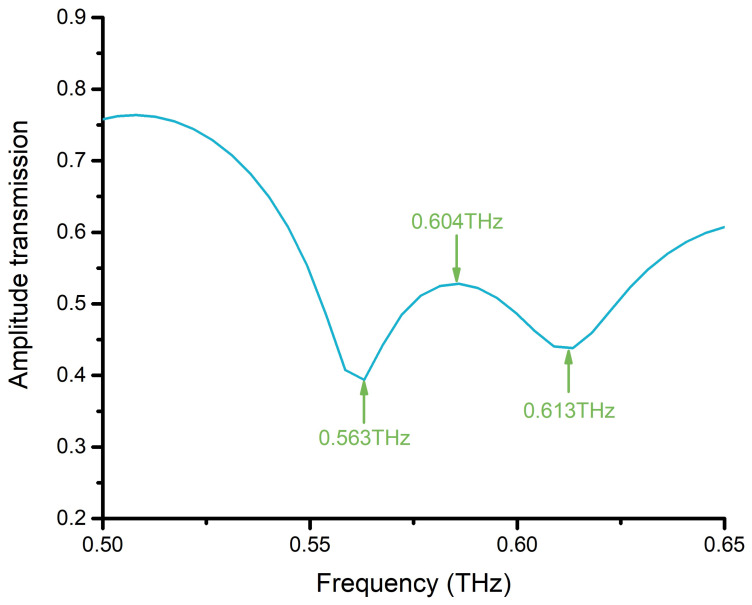
The amplitude transmission of the bare metamaterial sensor CWSRRs.

We measured the amplitude transmission of the bare metamaterial sensor CWSRRs, as illustrated in Figure 9. The resonance dips were at 0.563 THz and 0.613 THz, which was in good agreement with the simulation.

**Figure 10:**
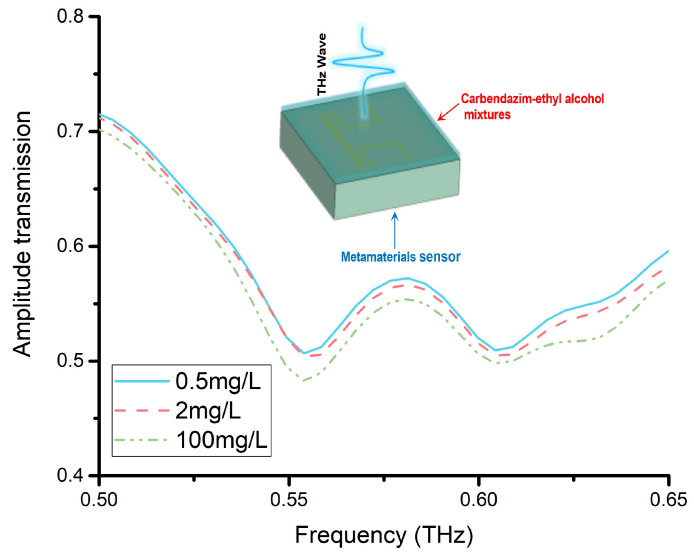
The amplitude transmission of the carbendazim–ethyl alcohol mixtures doped on CWSRRs.

Figure 10 shows the amplitude transmission of the carbendazim–ethyl alcohol mixtures doped on CWSRRs. The two resonance dips gradually shifted to a lower frequency than without the mixture on the sensor. When the carbendazim concentration was 100 mg/L, the two resonance dips were at 0.554 THz and 0.604 THz. The variation in carbendazim concentration caused the permittivity of the carbendazim–ethyl alcohol mixtures to change. For the permittivity of the 2 mg/L sample and 0.5 mg/L sample, the difference was not significant due to the carbendazim concentration being similar. When the carbendazim concentration was 2 mg/L, the two resonance dips were at 0.557 THz and 0.605 THz. Additionally, when the carbendazim concentration was 0.5 mg/L, the two resonance dips were at 0.556 THz and 0.605 THz. Thus, when the carbendazim concentration was down to 2 mg/L and 0.5 mg/L, the resonance dips were almost the same. It was difficult to tell one from the other. Using the CWSRRs, the carbendazim limit of detection was 100 mg/L.

**Figure 11:**
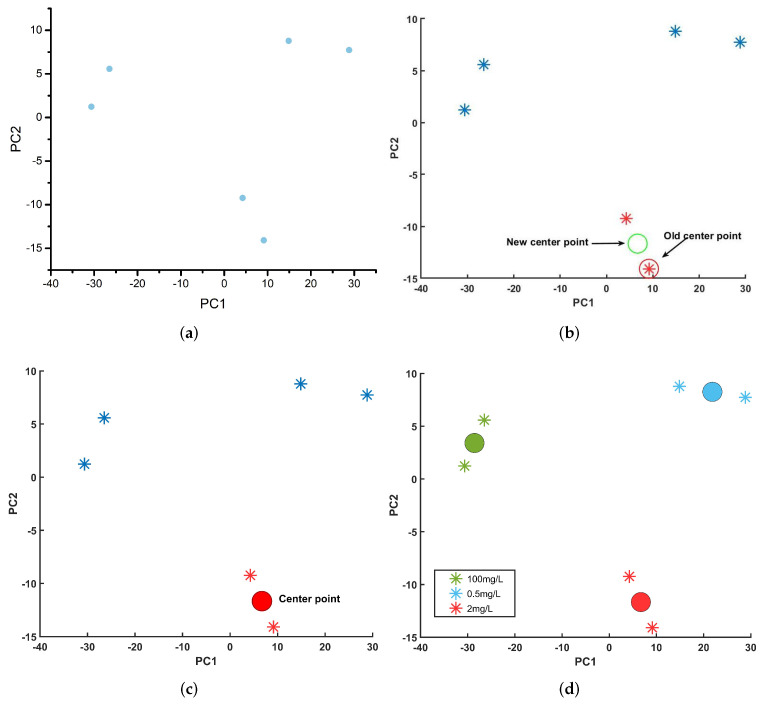
The result of PCA and mean shift execution process (**a**): PCA; (**b**) find a new center point; (**c**) discover the center of a cluster; (**d**) the result of mean shift.

Additionally, we used the mean shift unsupervised learning method to raise the minimum detectable concentration on the CWSRRs for metamaterial sensors. A total of six amplitude transmission spectrum samples for three carbendazim concentrations (0.5 mg/L, 2 mg/L, 100 mg/L) were analyzed. Before applying the mean shift, we reduced the dimension of the amplitude transmission spectra using principal component analysis (PCA). We discovered that the cumulative variance contribution rate of the first two principal components (PCs) was 96.1%. Usually, the original dataset may be replaced when the cumulative variance contribution rate is greater than 85% [41]. Thus, we set the bandwidth to 15 and used the first two PCs as the input of the mean shift. After performing PCA, the six samples were found to be in a plane without overlapping, as shown in Figure 11a.

The mean shift execution process is shown in Figure 11b–d. Initially, a sample was randomly selected as a center point(old center point). Then, using the mean shift, the new center point was discovered (see Figure 11b). The center of a cluster was discovered when the mean shift satisfied the conditions $∥M_{h,G}\left(x\right)∥\leq \varepsilon$ (set ε as $10^{-45}$), as shown in Figure 11c. We repeated that process until all cluster centers had been discovered. Finally, the outcome of the mean shift is shown in Figure 11d. The dot indicates the cluster’s center point. Additionally, the samples are denoted by an asterisk. Three center points fall between the cluster samples. Thus, the six samples were split into three clusters in Figure 11d that correspond to values of 0.5 mg/L, 2 mg/L, and 100 mg/L, respectively. The group of samples in the upper-left corner of the plot had a carbendazim concentration of 100 mg/L. Another was the group of samples in the lower-right corner of the plot with a carbendazim concentration of 2 mg/L. The remaining samples, which had a carbendazim concentration of 0.5 mg/L, are situated in the plot’s upper-right corner. This indicates that the mean shift could identify the sample successfully. By combining the metamaterial sensor and mean shift, the carbendazim limit of detection was 0.5 mg/L. As mentioned above, the carbendazim limit of detection was 100 mg/L when only the metamaterial sensor was used. Our proposed method enhanced the detection sensitivity 200 times compared to the sensitivity achieved only using the metamaterial sensor.

We also contrasted our proposed approach with other ones from the literature. The method under comparison operated in the terahertz band. Table 1 displays the results of the comparison. Our proposed method yielded a 0.5 mg/L limit of detection for carbendazim. It outperformed comparable methods in the literature.

**Table 1:** The carbendazim limit of detection.

Method	Limit of Detection
Ma et al. Ref [28]	50% (Mass fraction)
Cao et al. Ref [26]	2.5% (Mass fraction)
Qin et al. Ref [29]	2.5% (Mass fraction)
Yang et al. Ref [37]	2% (Mass fraction)
Qin et al. Ref [33]	5 mg/L
Nie et al. Ref [42]	10 mg/L
**Our proposed method**	0.5 mg/L

## 4. Conclusions

In this study, we combined a metamaterial sensor and mean shift to implement highly sensitive detection of carbendazim. The simulation and experiment results demonstrate that our proposed method could effectively detect carbendazim. The achieved carbendazim limit of detection was 0.5 mg/L; thus, sensitivity was improved by a factor of 200 times compared to the case of using the metamaterial sensor only. Since our method was based on measuring the change in the dielectric properties of the sample, it could be universally used to detect other benzimidazole fungicides. Nevertheless, the precision and accuracy of the test results were extremely sensitive to the deposition of nontarget substances. Selectivity should be considered in our future work. To implement selectivity, we will concentrate on preventing nontarget substances from contaminating the metamaterials during sample preparation and measurement.

## Data Availability

The data presented in this study are available on request from the corresponding author. The data are not publicly available due to privacy.

## References

[1] Kara M., Oztas E., Ramazanoullar R., Kouretas D., Veskoukis A.S. (2020). Benomyl, a benzimidazole fungicide, induces oxidative stress and apoptosis in neural cells. Toxicol. Rep..

[2] Aire T.A. (2005). Short-term effects of carbendazim on the gross and microscopic features of the testes of Japanese quails (Coturnix coturnix japonica). Anat. Embryol..

[3] Zq A., Yj A., Hp A., Jl A., St B., Pm A., Xw A., Ds A., Ying S.A. (2020). MIL-101(Cr)/MWCNTs-functionalized melamine sponges for solid-phase extraction of triazines from corn samples, and their subsequent determination by HPLC-MS/MS—ScienceDirect. Talanta.

[4] Attig J.B., Latrous L., Zougagh M., Rios N. (2021). Ionic liquid and magnetic multiwalled carbon nanotubes for extraction of N-methylcarbamate pesticides from water samples prior their determination by capillary electrophoresis. Talanta.

[5] Harshit D., Charmy K., Nrupesh P. (2017). Organophosphorus pesticides determination by novel HPLC and spectrophotometric method. Food Chem..

[6] Barbieri M.V., Postigo C., Guillem-Argiles N., Monllor-Alcaraz L.S., Simionato J.I., Stella E., Barcelo D., Miren L. (2019). Analysis of 52 pesticides in fresh fish muscle by QuEChERS extraction followed by LC-MS/MS determination. Sci. Total Environ..

[7] Qu F., Lin L., Cai C., Chu B., Nie P. (2020). Terahertz fingerprint characterization of 2,4-dichlorophenoxyacetic acid and its enhanced detection in food matrices combined with spectral baseline correction. Food Chem..

[8] Yao T., Liu A., Liu Y., Wei M., Liu S. (2019). Ratiometric fluorescence sensor for organophosphorus pesticide detection based on opposite responses of two fluorescence reagents to MnO2 nanosheets. Biosens. Bioelectron..

[9] Tao Z., Deng J., Wang Y., Chen H., Wang M. (2019). Competitive immunoassay for simultaneous detection of imidacloprid and thiacloprid by upconversion nanoparticles and magnetic nanoparticles. Environ. Sci. Pollut. Res..

[10] Fra C., Alo B., Naab B., Mmea C., Cva C. (2020). Bioactive microfluidic paper device for pesticide determination in waters. Talanta.

[11] Verma N., Bhardwaj A. (2015). Biosensor Technology for Pesticides-A review. Appl. Biochem. Biotechnol..

[12] Zhu J., Sharma A.S., Xu J., Xu Y., Chen Q. (2021). Rapid on-site identification of pesticide residues in tea by one-dimensional convolutional neural network coupled with surface-enhanced Raman scattering. Spectrochim. Acta Part A Mol. Biomol. Spectrosc..

[13] Zhu J., Ahmad W., Xu Y., Liu S., Chen Q., Hassan M.M. (2018). Development of a novel wavelength selection method for the trace determination of chlorpyrifos on Au@Ag NPs substrate coupled surface-enhanced Raman spectroscopy. Analyst.

[14] Lu Y., Li X., Li W., Shen T., Liu F. (2021). Detection of chlorpyrifos and carbendazim residues in the cabbage using visible/near-infrared spectroscopy combined with chemometrics. Spectrochim. Acta Part A Mol. Biomol. Spectrosc..

[15] Mikac L., Kovaevi E., Uki S., Rai M., Ivanda M. (2021). Detection of Multi-class Pesticide Residues with Surface-Enhanced Raman Spectroscopy. Spectrochim. Acta Part A Mol. Biomol. Spectrosc..

[16] Pham T.B., Hoang T., Pham V.H., Nguyen V.C., Nguyen T.V., Vu D.C., Pham V.H., Bui H. (2019). Detection of Permethrin pesticide using silver nano-dendrites SERS on optical fibre fabricated by laser-assisted photochemical method. Sci. Rep..

[17] Zhang Q., Li D., Cao X., Gu H., Deng W. (2019). Self-Assembled Microgels Arrays for Electrostatic Concentration and Surface-Enhanced Raman Spectroscopy Detection of Charged Pesticides in Seawater. Anal. Chem..

[18] Gong X., Tang M., Gong Z., Qiu Z., Wang D., Fan M. (2019). Screening pesticide residues on fruit peels using portable Raman spectrometer combined with adhesive tape sampling. Food Chem..

[19] Madianos L., Skotadis E., Patsiouras L., Filippidou M.K., Chatzandroulis S., Tsoukalas D. (2018). Nanoparticle based gas-sensing array for pesticide detection. J. Environ. Chem. Eng..

[20] Chen J., Huang M., Kong L. (2020). Flexible Ag/Nanocellulose fibers SERS substrate and its applications for in-situ hazardous residues detection on food. Appl. Surf. Sci..

[21] Xu W., Wang S., Li W., Zhang Z., Wang Y., Yang Y., Zhang H., Liu P., Xie L., Ying Y. (2022). Pesticide detection with covalent-organic-framework nanofilms at terahertz band. Biosens. Bioelectron..

[22] Tu S., Wang Z., Zhang W., Li Y., She Y., Du H., Yi C., Qin B., Liu Z. (2022). A new technology for rapid determination of isomers of hydroxybenzoic acid by terahertz spectroscopy. Spectrochim. Acta Part A Mol. Biomol. Spectrosc..

[23] Li F., Zhang J., Wang Y. (2022). Vibrational spectroscopy combined with chemometrics in authentication of functional foods. Crit. Rev. Anal. Chem..

[24] Huo Z., Zhi L.I., Tao C., Qin B. (2017). Quantitative determination of Auramine O by terahertz spectroscopy with 2DCOS-PLSRmodel. Spectrochim. Acta A Mol. Biomol. Spectrosc..

[25] Chen Q., Jia S., Qin J., Du Y., Zhao Z. (2020). A Feasible Approach to Detect Pesticides in Food Samples Using THz-FDS and Chemometrics. J. Spectrosc..

[26] Cao B., Li H., Fan M., Wang W., Wang M. (2018). Determination of pesticides in a flour substrate by chemometric methods using terahertz spectroscopy. Anal. Methods.

[27] Liu L., Hu P., Yang F., Song M. (2020). Application of terahertz time-domain spectroscopy combined with support vector machine to determine tea and pesticide samples. Mater. Express.

[28] Ma Y., Qiang W., Wang X., Wang H. (2011). Research of pesticide residues on fruit by terahertz spectroscopy technology. Proc. SPIE-Int. Soc. Opt. Eng..

[29] Qin B., Li Z., Luo Z., Li Y., Zhang H. (2017). Terahertz time-domain spectroscopy combined with PCA-CFSFDP applied for pesticide detection. Opt. Quantum Electron..

[30] Xu W., Xu W., Xie L., Xie L., Ying Y., Ying Y. (2017). Mechanisms and applications of terahertz metamaterial sensing: A review. Nanoscale-Cambridge.

[31] Xie L., Gao W., Shu J., Ying Y., Kono J. (2015). Extraordinary sensitivity enhancement by metasurfaces in terahertz detection of antibiotics. Sci. Rep..

[32] Qin J., Xie L., Ying Y. (2016). A high-sensitivity terahertz spectroscopy technology for tetracycline hydrochloride detection using metamaterials. Food Chem..

[33] Qin B., Li Z., Hu F., Hu C., Chen T., Zhang H., Zhao Y. (2018). Highly Sensitive Detection of Carbendazim by Using Terahertz Time-Domain Spectroscopy Combined With Metamaterial. IEEE Trans. Terahertz. Sci. Technol..

[34] Yang S., Li C., Mei Y., Liu W., Xu K. (2021). Determination of the Geographical Origin of Coffee Beans Using Terahertz Spectroscopy Combined With Machine Learning Methods. Front. Nutr..

[35] Silva V.H.D., Vieira F.S., Rohwedder J.J.R., Pasquini C., Pereira C.F. (2017). Multivariate quantification of mebendazole polymorphs by terahertz time domain spectroscopy (THz-TDS). Analyst.

[36] Wang B., Qin X., Meng K., Zhu L., Li Z. (2022). Classification of Amino Acids Using Hybrid Terahertz Spectrum and an Efficient Channel Attention Convolutional Neural Network. Nanomaterials.

[37] Yang R., Li Y., Qin B., Zhao D., Gan Y., Zheng J. (2022). Pesticide detection combining the Wasserstein generative adversarial network and the residual neural network based on terahertz spectroscopy. RSC Adv..

[38] Fukunaga K., Hostetler L. (1975). The estimation of the gradient of a density function, with applications in pattern recognition. IEEE Trans. Inf. Theory.

[39] Cheng Y. (1995). Mean shift, mode seeking, and clustering. IEEE Trans. Pattern. Anal. Mach. Intell..

[40] O’Hara J.F., Singh R., Brener I., Smirnova E., Zhang W. (2008). Thin-film sensing with planar terahertz metamaterials: Sensitivity and limitations. Opt. Express.

[41] Chen T., Li Z., Mo W. (2013). Identification of biomolecules by terahertz spectroscopy and fuzzy pattern recognition. Spectrochim. Acta Part A Mol. Biomol. Spectrosc..

[42] Nie P., Qu F., Lin L., He Y., Feng X., Yang L., Gao H., Zhao L., Huang L. (2021). Trace Identification and Visualization of Multiple Benzimidazole Pesticide Residues on Toona sinensis Leaves Using Terahertz Imaging Combined with Deep Learning. Int. J. Mol. Sci..

